# Complete Genome Sequence of Classical Swine Fever Virus Isolated near the Demilitarized Zone in the Republic of Korea

**DOI:** 10.1128/genomeA.00145-17

**Published:** 2017-04-06

**Authors:** Sok Song, SeEun Choe, RaMi Cha, Ki-Sun Kim, In-Soo Cho, Dong-Jun An

**Affiliations:** Viral Disease Division, Animal and Plant Quarantine Agency, Gimcheon, Gyeongbuk, Republic of Korea

## Abstract

The YC16CS (genotype 2.1) strain of classical swine fever virus, isolated from infected pigs in Yeoncheon province, Republic of Korea, near the demilitarized zone, has a high identity with the PC11WB strain of the virus. This is significant in that it is the first case of transmission from wild boars to breed pigs revealed by an epidemiological investigation.

## GENOME ANNOUNCEMENT

Classical swine fever (CSF) is a highly contagious fatal disease of pigs and wild boars caused by the CSF virus (CSFV), a pestivirus belonging to the family *Flaviviridae* (1). The CSFV genome is approximately 12.3 kb in length, and it comprises a single long open reading frame (ORF). In this study, we report the complete genome sequence of CSFV strain YC16CS, which was isolated from a pig farm in Yeoncheon province, Republic of Korea, in 2016. The YC16CS-infected pigs strongly showed typical symptoms of CSFV (high fever, lack of appetite, conjunctivitis, and constipation) (2).

Total RNA was extracted using the RNeasy minikit (Qiagen, USA), and cDNA was synthesized with HelixCript (NANOHELIX, Republic of Korea) using random hexamer oligo primers. The overlapping genome fragments were amplified from the cDNA using the Qiagen HotStartaq Master Mix kit (Qiagen, USA) (3), and the PCR products were sequenced with an ABI Prism 3730xi DNA sequencer. The generated sequences were assembled and aligned using the BioEdit program, and the phylogenetic tree was analyzed using the Mega6.1 program (4). The complete sequence of YC16CS covered the entire length of the 12,297 nucleotides (nt), including a 374-nt 5′ untranslated region (UTR), an 11,696-nt ORF encoding a 3,898-amino acid-long polyprotein, and a 227-nt 3′ UTR. The maximum likelihood tree, which included the 12 CSFV complete genome sequences available in GenBank, showed that the nucleotide sequences were 94 to 97.9% for subgenotype 2.1, 90.8% for subgenotype 2.2, and 88.5 to 89.3% for subgenotype 2.3. The comparative analysis of gene nucleotide sequences of strain YC16CS with those of the reference strain PC11WB (a subgenotype 2.1 virus isolated from Korean wild boar) (5), revealed high sequence identities: 97.8% for the Npro genes, 96.4% for the C genes, 97.2% for the Erns genes, 97.5% for the E1 genes, 97.6% for the E2 genes, 98.5% for the P7 genes, 96.9% for the NS2 genes, 97.3% for the NS3 genes, 98.9% for the NS4A genes, 98.0% for the NS4B genes, 97.3% for the NS5A genes, and 97.5% for the NS5B genes. A similar analysis of 75 complete CSFV genome sequences deposited in GenBank revealed that the YC16CS strain showed 97.9% sequence homology at the nucleotide level with the strain CSFV/Mongolia/Bu08/2014 (accession no. LC086647) which was isolated in Mongolia in 2014.

However, as a result of the epidemiological investigation for CSFV YC16CS outbreak, there was no evidence of domestic inflow from other countries. Interestingly, the YC16CS strain has a very different nucleotide sequence with CSFV circuiting among pig farms but has a high similarity with the PC11WB strain isolated from wild boars in 2011 (5).

Yeoncheon province, where the YC16CS strain was isolated, is located near the demilitarized zone of North Korea. Also, the CSFV antibodies have been detected frequently in wild boars living in this region every year. Therefore, from a comprehensive point of view, it appears that wild boars transmitted the YC16CS strain to breed pigs.

### Accession number(s).

The complete genome sequence of the YC16CS strain has been deposited in GenBank under the accession number KY290453.
